# The value of person-first language in forensic mental health services

**DOI:** 10.1192/bjb.2026.10230

**Published:** 2026-08

**Authors:** Jack Tomlin, Asya Mitisheva, Sarah Kilbane, Sarah Markham, John Tully

**Affiliations:** 1 School of Medicine, University of Warwick, Coventry, UK; 2 School of Law and Criminology, University of Greenwich, London, UK; 3 Institute of Psychiatry, Psychology and Neuroscience, King’s College London, London, UK; 4 Institute of Mental Health, University of Nottingham, Nottingham, UK

**Keywords:** Forensic psychiatry, stigma and discrimination, person-first language, labels, discrimination

## Abstract

Labels in healthcare influence perceptions, treatment and stigma. Although they can aid diagnosis and guide care, pejorative labels can dehumanise and perpetuate prejudice. Person-first language, which foregrounds the individual rather than a condition or behaviour, has been linked to reduced blame, stigma and social distance across mental health and forensic populations. Evidence suggests it may foster empathy, recovery-oriented attitudes and rehabilitation, while challenging essentialist and pessimistic views of mental illness and offending. Limitations include minimising structural coercion and lived experience. This editorial explores the evidence, mechanisms and practical applications of person-first language in forensic mental health, advocating cautious implementation and further research.

Labels used in healthcare contexts matter. Labels can influence how people are viewed and treated by others. They can be helpful in supporting diagnosis, indicating needs and signposting treatments. But labels can also be harmful. Consider the use of ‘psycho’ or ‘invalid’ as instruments to diminish, hurt and dehumanise. Their impact and significance is evidenced by the efforts of advocacy groups to reclaim pejorative terms and denude them of their hurtfulness. For example, MAD activists have embraced ‘mad’ and ‘lunatic’ to celebrate the difference and overcome the prejudice assigned to mental illness.

In few areas of healthcare is the debate over labels more confused or contentious than in forensic mental health services. Owing to their interaction with both healthcare services and the criminal justice system, individuals receiving involuntary treatment in forensic services may have multiple labels applied to them. Like people under the care of general mental health services, they may be variously referred to as ‘patients’, ‘service users’ and ‘clients’, but also as ‘offenders’, ‘mentally disordered offenders’ and, in some cases, ‘prisoners’. Further, they may be subjected to the same range of labels used to describe mental disorders in the general population, including ‘schizophrenic’, ‘manic–depressive’ and ‘psychotic’.

Throughout mental healthcare, there is a growing appetite for person-first language. Person-first language places primacy on a person’s identity as a human being, rather than a particular condition or past behaviour, for example ‘person with schizophrenia’ rather than ‘schizophrenic’, or ‘person with a substance use disorder’ rather than ‘drug user’. It means avoiding language that describes people by a specific characteristic, trait or diagnosis that might carry stereotyped and prejudiced associations. Its adoption has been advocated by organisations in multiple countries, including Mind UK,^1^ the US National Institute on Drug Abuse^2^ and the Mental Health Commission of Canada.^3^

This editorial explores the evidence base for person-first language, its limitations and its potential application in forensic mental health settings.

## What evidence is there for person-first language use?

Person-first language has been most commonly studied using cross-sectional survey designs that evaluate respondents’ attitudes towards individuals with specific conditions or traits, and the potential impact of the use of terminology. For instance, two studies^4,5^ found that labelling individuals as ‘people with substance use disorders’ instead of ‘substance abusers’ was associated with reduced blame, and less punitive and more compassionate and rehabilitative attitudes, in both the public and professional samples (with small to medium effect sizes). Similarly, a randomised mixed-methods experiment conducted using two surveys with a US nationally representative sample (*n* = 2800) found that person-first language in media reportage (e.g. ‘person with a felony conviction’ versus ‘felon’) was linked to fewer negative associations (e.g. ‘dangerous’, ‘serious criminal’) and more positive associations (e.g. ‘needs rehabilitation’, ‘made a mistake’), with 68% of participants in the criminal labels condition choosing negative associations, compared with 50% in the person-first condition.^6^ One US-based study (*n* = 701)^7^ found that changing the term ‘the mentally ill’ to ‘people with mental illness’ was associated with less authoritarian and socially restrictive attitudes among students and counselling professionals in training (both with medium effect sizes). Recently, in a randomised study (*n* = 668), we reported that ‘person working towards recovery using forensic mental health services’ was linked to lower desire for social distance and higher perceptions of responsibility for behaviour in a UK nationally representative sample than ‘forensic mental health patient’ or ‘mentally disordered offender’ (small to medium effects).^8^ A study involving members of the US general public (*n* = 1015) found that describing someone as a ‘person who was formerly incarcerated’, rather than an ‘ex-convict’, was linked to statistically significantly lower negative stereotypes and desired social distance scores, which indirectly increased support for reintegration.^9^ These findings, although mostly cross-sectional, demonstrate a consistent link between person-first language and less stigmatising attitudes.

## Why does person-first language influence social attitudes?

The stigma-reducing potential of person-first language might be explained by several interconnected mechanisms. The first of these relates to the use of labels. Labels applied by those in power can reinforce existing power dynamics, for example by denoting the patient as inferior.^10^ This can perpetuate institutional harms. For those who are labelled, such terms can cause or exacerbate the internalisation of stigma and can lead to stereotype-consistent actions through self-fulfilling prophecy.^11^ In an effort to avoid potentially damaging labels, individuals may avoid treatment linked to identifying language, which can lead to poorer outcomes.^12^ This can not only influence the course of an illness, but may also have an impact on important areas like social inclusion and employment. Instead, person-first language places the ‘person’ before any other descriptors of a condition^13^ or deviant behaviour,^11^ helping to reduce the weight that may come with such labels. If stigma reduces a person ‘from a whole and usual person to a tainted, discounted one’,^14^ then person-first language may encourage considering the ‘whole’ of the individual.

Second, language that centres the presence of a mental health diagnosis (e.g. ‘schizophrenic’) or the commission of a crime (e.g. ‘offender’) can be seen as essential to a person’s character. Such ‘inherentness’ can make it seem as though the person is one with the label, creating a challenge in separating the labelled person from the stigma associated with a behaviour or trait and reinforcing a sense of ‘otherness’. This ‘essentialism’ has been identified in relation to both mental illness^15^ and criminal justice involvement,^16^ and can play a role in how social categories are developed. Although identity-first language may be preferred by some community members because of its ability to create belongingness among group members (for instance, stronger group identity has been found to predict favourability of identity-first language),^17^ the labels that come with such language may also increase psychological distance between persons outside of and within these groups. For people on the outside of the group, identity-first language can dehumanise. Person-first language, on the other hand, prioritises humanity. This may reduce the psychological distance between the subject of the label and the observer, leading to more empathetic views and an increased desire to engage in helping behaviour. Indeed, initiatives that focus on humanising (or ‘rehumanising’) approaches have been found to reduce stigma.^18,19^

Third, essentialism has been linked to pessimism regarding treatability.^20^ Person-first language challenges ‘prognostic pessimism’ through language that is ‘recovery-oriented’, prioritising dignity, respect and individual strengths and needs.^13^ Such language allows non-group members to see that the label is not static but dynamic, with the potential to change over time with suitable intervention. This may then empower individuals towards agency over their own care. Such language has been linked to the endorsement of more rehabilitative care from mental health professionals,^5^ with perceived treatability also leading to greater social acceptance.^15^ Perhaps most importantly for those who, through or in the context of experiencing a mental disorder, have offended and caused (sometimes irrevocable) harm to others, person-first language emphasises their agency and accountability. This self-acknowledgement can be crucial if individual patients are to take responsibility for themselves and their actions in the future and optimise their potential to adaptively reintegrate into society and work towards self-actualisation.

## What are the limitations of person-first language?

It is possible that the adoption of person-first language could downplay the coercive and involuntary nature of forensic services by emphasising a degree of autonomy that patients do not have. Furthermore, some advocacy groups propose that person-first language minimises lived experiences (e.g. blindness), hides aspects of identity (e.g. autism) or may promote further stigmatisation by identifying certain characteristics as negative and thus something a person should seek distance from (e.g. we refer to doctors, not people who practise medicine).^21^

Some advocates within the neurodivergence and disability communities prefer identity-first language, claiming that it affirms pride and agency from within, rather than separating the person from their experience.^22^ Indeed, not all people with mental health conditions may find person-first or identity-first language activism helpful, preferring the use of more neutral and scientific terminology. This includes language emerging from the growth of computational psychiatry, which acts to bridge the divides between the various conceptual understandings of mental health conditions. Furthermore, victims of crime may understandably resist a linguistic approach that may be seen to minimise or de-emphasise the harms a person has committed.

## How can person-first language be incorporated into practice?

A change in language use does not necessarily avert stigma. Avoiding the term ‘schizophrenia’, for example, may be preferable to some, but without public education and other work to reduce broader stigma about severe mental illness, it is possible that any alternative term to describe the condition could then also become loaded with negative connotations. Nonetheless, incorporation into practice of person-first language by individuals and organisations may have a small but important impact.

As an example, new and amended mental health legislation and complementary codes of practice could replace ‘mentally disordered offender’ with ‘person in forensic mental health services’. Where more specificity is required, phrases such as ‘person under a section of Part III of the MHA [Mental Health Act]’ could be used. Information leaflets for family and carers, workshops and training sessions, and in-patient notes could also adopt person-first language. Researchers, professional bodies, academic journals and higher education providers can support this when publishing manuscripts and policy documents or developing and advertising curricula. ‘Mentally disordered offender’ could be amended in all materials published by the Royal College of Psychiatrists’ Faculty of Forensic Psychiatry to a more person-first term, for example. Media corporations should adopt policies to use person-first language to describe this population unless certain affected groups have argued against it or it is indicated otherwise (e.g. the *Guardian* newspaper’s style guide on identity-first language). Table 1 gives some suggested changes to language used in forensic mental health contexts.

**Table 1:** Person-first language recommendations in forensic mental health contexts Table 1 long description.

Sector/context	Terms to avoid	Recommended person-first alternatives	Rationale
Clinical/legal context	‘Mentally disordered offender’	‘Person in forensic mental health services’, ‘Person in forensic mental health services under section 37 of the Mental Health Act’	Maintains legal clarity while avoiding unnecessarily labelling
Mental health legislation, codes of practice, nursing notes and clinical discussions	‘Restricted/sectioned patient’, ‘detained’	‘Person under a section of Part III of the MHA’, ‘Person receiving support/treatment under the Mental Health Act’	Encourages precise, respectful terminology that does not reduce a person to a legal status or a diagnoses
Legal route into care	‘Detained patient’, ‘Sectioned patient’, ‘Offender under hospital order’	‘Person receiving compulsory forensic mental healthcare’, ‘Person subject to forensic mental healthcare’	Recognises the involuntary nature of the services without reinforcing criminalisation
Behavioural and risk descriptors	‘Non-compliant’, ‘Resistant’, ‘Dangerous/potentially violent patient’	‘Person not currently engaging with treatment’, ‘Person expressing mistrust’, ‘Person currently assessed as presenting risk’	Reduces pejorative tone, presents trauma-informed interpretation of behaviour, reflects temporality and contextual risk
Academic writing	‘Forensic mental health patient’	‘Person with criminal justice involvement and serious mental health needs’, ‘Person in forensic mental health services’	Maintains neutrality, models respectful language
Media reporting	‘Psychotic’, ‘schizophrenic’	‘Person in forensic mental health services’ or any of the above	Avoids sensation-seeking language and the creation of moral panics that would increase perceptions of risk and willingness for social distance

## Conclusion

Language can contribute to the development of stigma. Stigmatising attitudes can increase social exclusion, dehumanisation and distrust, all of which can worsen mental health and increase risk of offending. In contrast, careful and thoughtful use of person-first language can promote agency, better understanding and more reflective attitudes across professional services and the public domain. The data to support its use remains limited. However, based on some promising outcomes from initial work, we endorse its cautious adoption in forensic mental health practice. We also strongly advocate further empirical study of its impact, including any unwanted effects.

## About the authors

**Jack Tomlin** is an MBChB student at the School of Medicine, University of Warwick, Coventry, UK and an affiliate researcher in the School of Law and Criminology, University of Greenwich, London, UK. **Asya Mitisheva** is a graduate of the School of Law and Criminology, University of Greenwich, London, UK. **Sarah Kilbane** is a senior lecturer in the School of Law and Criminology, University of Greenwich, London, UK. **Sarah Markham** is a biostatistician in the Institute of Psychiatry, Psychology & Neuroscience, King’s College London, London, UK. **John Tully** is a Clinical Associate Professor in Forensic Psychiatry and a Medical Research Council (MRC) Clinician Scientist Fellow in the Institute of Mental Health, University of Nottingham, Nottingham, UK.

## Table 1 Long description

A table with five columns and six rows, including a header row. The columns are labeled Sector/context, Terms to avoid, Recommended person-first alternatives, and Rationale. The rows provide examples of terms to avoid and their recommended person-first alternatives in various contexts such as clinical/legal context, mental health legislation, legal route into care, behavioral and risk descriptors, academic writing, and media reporting. Each row includes the sector or context, the term to avoid, the recommended person-first alternative, and the rationale for the change.

Navigate back to Table 1.
